# Experiences of violence and the use of sleep-inducing medicines: a population-based study

**DOI:** 10.3389/fgwh.2026.1790824

**Published:** 2026-04-30

**Authors:** Franciéle Marabotti Costa Leite, Fernanda Garcia Gabira Miguez, Mariana Ketlin Monteiro Martins, Gabriela Oliveira, Isaura Barros Alves Pinto, Micael Franco Alves, Dherik Fraga Santos

**Affiliations:** 1 Postgraduate Program in Collective Health, Federal University of Espírito Santo, Vitória, Espírito Santo, Brazil; 2 Department of Epidemiological Analysis and Surveillance of Non-Communicable Diseases, Ministry of Health, Brasília, Federal District, Brazil; 3 Department of Medicine, Federal University of Catalão, Catalão, Goiás, Brazil

**Keywords:** domestic violence, gender-based violence, intimate partner violence, sleep aids pharmaceutical, women’s health

## Abstract

**Objective:**

This study aimed to verify the prevalence of sleep-inducing medicine use and its association with violence experiences among women.

**Methods:**

A cross-sectional study was conducted in 2022 in Vitória, Espírito Santo, Brazil, with 1,086 women aged 18 or older who had an intimate partner in the 24 months before the research. The outcome was the use of sleep-inducing medications and the independent variables was sociodemographic and economic characteristics, experiences of violence including, childhood sexual violence and intimate partner violence. We evaluated data descriptively, adopting 95% confidence intervals, Pearson's chi-square test or Fisher's exact test, and Poisson regression analysis.

**Results:**

The prevalence of lifetime use of sleep-inducing medicine was 37.2%, and the prevalence of current use was 18.4%. The lifetime or current use of sleep-inducing medicine was associated with age 60 years and above, not being in a marital relationship, having experienced sexual violence in childhood, and having suffered intimate partner violence (IPV) during their lifetime.

**Conclusion:**

We conclude that socioeconomic profile and exposure to violence are related to an increased odds of using sleep-inducing medicine.

## 1 Introduction

Violence against women is defined as any action or omission based on gender that causes death, injury, physical, sexual, or psychological distress, and moral or patrimonial damage. Depending on the several forms of abuse and subjugation endured, violence against women can be categorized as physical, psychological, sexual, economic, and moral violence (1). In 2023, a total of 302,856 reports of violence against women were recorded, a significant increase (40.2%) from the 216,024 cases in 2022. This growth may reflect both a real increase in cases and a greater capacity to recognize violence, as well as improved data collection (2).

We should consider that women who experience violence are frequently affected by feelings of anguish and loneliness, and may suffer adverse effects on their self-esteem, self-image, and quality of life (3). In this sense, female victims of violence typically develop conditions that affect their psychological well-being and have severe consequences, such as difficulties in daily life, removal from work, increased demand for health services, and increased odds of drug and medicine use (4, 5). These disorders impact individuals and generate considerable social and economic costs (6).

Sleep disorders are recurrent among the mental health complaints of women experiencing violence (7). Different studies reveal the detrimental effects of violence on women's sleep quality (8, 9). In this setting, we observe a more significant demand for pharmacological and non-pharmacological care to improve sleep quality (10). Considering that access to health services is lower for women in socially vulnerable situations, this situation can promote self-care actions without health professional guidance, including situations of self-medication with sleep-inducing medicines (11).

The World Health Organization (12) characterizes self-medication as a way of using or recommending a medicine based on one's own experience, without instruction from a qualified professional. The most commonly used medicines for treating insomnia are benzodiazepines, melatonin, herbal remedies, and teas. Self-medication with unprescribed prescription drugs poses a potential risk of increased undesirable side effects (13). Conversely, sleep-inducing medicines, when used appropriately and under the supervision of healthcare professionals, are of great value in the care of individuals with sleep disorders or other mental health conditions (14). However, the increasing use of these medicines is a Public Health problem and requires professionals to pay closer attention to the care of women (15).

In this context, the use of sleep-inducing medications remains poorly explored in women's health, particularly regarding its relationship with experiences of violence, and is insufficiently addressed in developing countries. This study may contribute to a better understanding of coping strategies and support the development of public health actions targeting women in vulnerable situations. Therefore, the present study aims to verify the prevalence of the use of sleep-inducing medicines and its association with violence against women.

## 2 Methods

### 2.1 Study design and context

Cross-sectional, quantitative, analytical study, with population-based data, conducted from January to May 2022, with a pilot study conducted in December 2021.

The study was conducted in Vitória, capital of the state of Espírito Santo, Brazil. The municipality has a population of 327,801 people, as indicated in the 2010 Census of the Brazilian Institute of Geography and Statistics (IBGE), with a territorial extension of 97.12 km^2^, with a per capita GDP of BRL 85,035.67. The census recorded 196,018 females, and 155,673 women were aged 18 and above (16).

### 2.2 Sample

In total, 1,086 women aged 18 or older who reported having an intimate partner in the 24 months before data collection comprised the sample for this study. The exclusion criterion was based on intellectual or sensory conditions that could compromise women's understanding or communication, preventing them from responding to the research data collection instruments. An intimate partner was defined as someone who, regardless of a formal relationship (partners, former partners, boyfriends, or common-law marriage), was still having sexual intercourse with the woman (17).

### 2.3 Sample calculation

A multistage sampling strategy was employed. The primary sampling units were the census tracts of the municipality of Vitória, as defined by the 2010 Census of the Brazilian Institute of Geography and Statistics (IBGE) (17). The total number of households in the urban area of Vitória, Espírito Santo, in 2010 was divided by 100 (the number of sectors to be visited) to determine the sampling interval, maintaining proportionality to the number of households and women in each sector. The tracts were then ordered according to socioeconomic level, and the number 513 was randomly selected using the R statistical software, identifying the first sector. The remaining sectors were selected by successively adding the sampling interval to the initial sector until the entire list was covered.

Following the selection of census tracts, households were randomly chosen from the list available on the IBGE online platform (17). In each selected household, a list of eligible women—those meeting the study's inclusion criteria—was compiled, and one woman was then randomly selected to participate in the interview. We calculated the sample size as per the estimated prevalence of intimate partner violence in the study population, considered at 50% and a 95% confidence interval, with an acceptable error of 5%. Thus, to enable the study of the associations of risk factors, we maintained the 95% CI, with 80% power and a 1:1 exposed/unexposed ratio. Then, the value obtained was increased by 10% to account for losses and 30% for confounding factors, resulting in a sample size of 1,100 women (17).

### 2.4 Data collection

Trained and supervised teams collected data in the field. All interviews were conducted in person by female interviewers after the participants signed an explicit informed consent form and were informed about the anonymity and assurance of the confidentiality of the interviews, which were conducted in the participant's own residence in a reserved, private location with only the woman and the interviewer, and lasting an average of 30 min. The data were collected on tablets with management of the Research Electronic Data Capture (REDCap) electronic data capture tool (17).

### 2.5 Study variables

#### 2.5.1 Outcome

The use of sleep-inducing medicines during lifetime was dichotomized (yes/no) based on the question “Have you ever used sleeping pills/sleep-inducing medicines?”, and current use through “Do you currently use sleeping pills/sleep-inducing medicines?”, with examples including controlled medicines (amitriptyline, nortriptyline, clonazepam, diazepam, zolpidem, zopiclone, melatonin, and trazodone). We also considered home-based remedies (extracted from different plants) and homeopathic medicines made with substances extracted from the plant, animal, and mineral kingdoms (gelsemium sempervirens, nux vomica, lachesis mutus, arsenicum album, sulfur, and natrum muriaticum).

#### 2.5.2 Independent variables

The categorical sociodemographic and economic independent variables are composed of age group (18–39 years; 40–59 years; ≥60 years), ethnicity/skin color (white people; black people; brown people; other), with the category “other” corresponding to the grouped ethnicity/skin color of yellow and Indigenous people, schooling (0–8 and ≥9 study years), household income by tercile (first; second; third), in a relationship (yes; no), where those who reported being married, in common-law marriage, or dating were considered as “Yes”.

Regarding experiences of violence, childhood sexual violence was ascertained through women's self-report and categorized as “Yes” or “No”, while intimate partner violence during lifetime and the COVID-19 pandemic was screened through women's report when responding to the World Health Organization instrument named WHO VAW Study, a questionnaire validated in Brazil (18), which can assess whether the woman was a victim of intimate partner violence through thirteen questions, with six questions referring to physical violence, four to psychological violence, and three to sexual violence perpetrated against women in different social contexts (19). For this purpose, we categorized the score obtained as “Yes”, considering any affirmative response to one of the types of violence. “No” resulted in two categorical binary variables, namely, one referring to IPV during lifetime and the other during the COVID-19 pandemic.

Furthermore, to construct the variable related to the number of intimate partner violence (IPV) types suffered during lifetime and the pandemic, we considered the three violence types (physical, psychological, and sexual). We created two distinct, categorical variables, composed of four levels: “no violence”, “one type”, “two types”, and “three types” of IPV, as per the number of modalities reported by each participant.

### 2.6 Data analysis

The data were first evaluated by describing absolute and relative frequencies and 95% confidence intervals. This was followed by bivariate analysis using Pearson's chi-square (*χ*^2^) test or Fisher's exact test, as appropriate. We performed a multivariate analysis using Poisson regression with robust variance to estimate the prevalence ratio, where adjustments were considered for *p*-values less than or equal to 0.20, and retained when significance was 5%. The data were analyzed using Stata 17.0 software.

### 2.7 Analytical conceptual model

**Figure 1:**
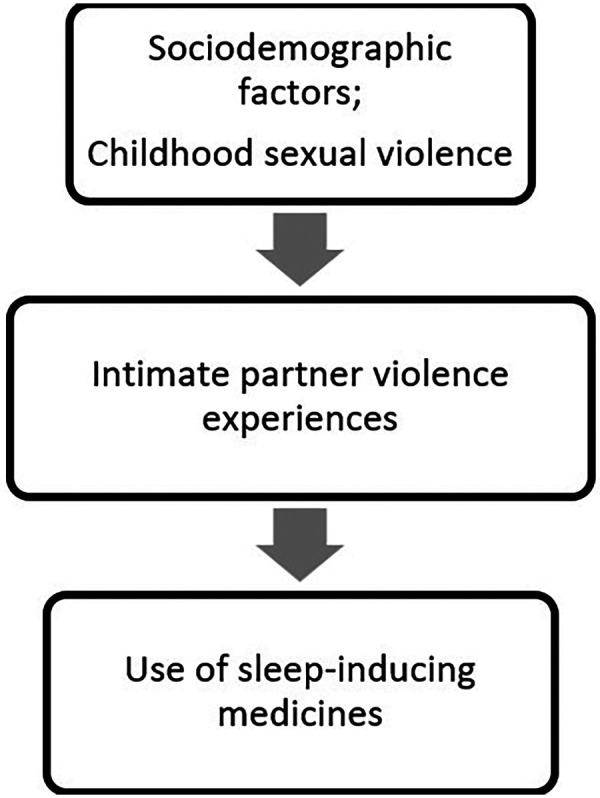
Hierarchical model of the relationships between socioeconomic characteristics and experience of violence and the use of sleep-inducing medicines among women residing in Vitória, Espírito Santo, Brazil.

Data analysis adjustment followed the conceptual model in Figure 1, which was developed based on a literature review regarding factors that may influence the use of sleep-inducing medicines. Thus, the adjustment followed a hierarchical order of variables, contributing to coherence in the analytical process. Notable are four distinct variables regarding the experience of violence (lifetime IPV, IPV during the pandemic, and the number of IPV experiences during lifetime and the pandemic). Therefore, the adjustment described in the figure was performed separately for each IPV variable.

### 2.8 Ethical aspects

The study was approved in April 2021 by the Ethics Committee under the Certificate of Submission for Ethical Review, number 41628820 and Certificate of Presentation for Ethical Review (CAAE) number 41628820.6.0000.5060. All interviewees signed the informed consent form and the study's objectives and its possible risks and benefits were clarified. The interviews took place in a private environment and confidentiality was guaranteed.

## 3 Results

The prevalence of lifetime use of sleep-inducing medicines among women residing in Vitória was 37.2% (95% CI 34.4–40.1), and current use was 18.4% (16.2–20.8), as shown in Table 1.

**Table 1:** Prevalence of current and lifetime use of sleep-inducing medicines among women residing in Vitória, Espírito Santo, Brazil (*N* = 1,086).

Characteristic Sleep-inducing medicine use	Category	*n*	Percentage (%)	95% confidence interval (95% CI)
Lifetime use	No	682	62.8	59.9–65.6
	Yes	404	37.2	34.4–40.1
Current use^a^	No	883	81.6	79.2–83.8
	Yes	199	18.4	16.2–20.8

a 4 missing.

Table 2 shows that the lifetime use of sleep-inducing medicines was related to age group, childhood sexual violence, lifetime experience of intimate partner violence (IPV), and IPV during the pandemic, as well as the amount of lifetime IPV. Regarding current use, a relationship was observed with age group, ethnicity/skin color, lifetime experience of IPV, and the amount of IPV experienced during lifetime (*p* < 0.05).

**Table 2:** Women's lifetime and current use of sleep-inducing medicines according to sociodemographic characteristics and experiences of violence. Vitória, Espírito Santo, Brazil, 2022.

Variable	Category	*Lifetime use*			*Current use*		
		N (%)	95% confidence interval (95% CI)	*p-value*	N (%)	95% CI	*p-value*
Age group (years)	18–39	121 (29.8)	25.5–34.4	<0.001	44 (10.8)	8.2–14.3	<0.001
	40–59	166 (39.7)	35.1–44.5		81 (19.4)	15.9–23.5	
	60 and above	117 (44.7)	38.7–50.7		74 (28.6)	23.4–34.4	
Ethnicity-Skin color	White people	176 (40.4)	35.9–45.0	0.077	103 (23.7)	19.9–27.9	0.002
	Black people	79 (40.3)	33.7–47.3		34 (17.4)	12.7–23.4	
	Brown people	143 (33.2)	28.9–37.8		59 (13.7)	10.8–17.3	
	Other	06 (26.1)	12.2–47.3		03 (13.0)	4.3–33.6	
Schooling (years)	≤8 years	71 (35.0)	28.7–41.8	0.467	41 (20.1)	15.2–26.3	0.461
	≥9 years	333 (37.7)	34.6–41.0		158 (17.9)	15.6–20.7	
Income tertile	First	150 (36.2)	31.7–41.0	0.874	72 (17.4)	14.0–21.4	0.800
	Second	132 (37.8)	32.9–43.0		66 (19.0)	15.2–23.5	
	Third	122 (37.7)	32.6–43.2		61 (19.0)	15.1–23.7	
In a relationship	Yes	320 (36.1)	33.0–39.3	0.120	159 (17.7)	15.3–20.4	0.209
	No	84 (42.0)	35.3–49.0		43 (21.5)	16.3–27.7	
Childhood sexual violence	No	345 (35.5)	32.6–38.6	0.001	172 (17.8)	15.5–20.3	0.136
	Yes	59 (51.3)	42.2–60.3		27 (23.5)	16.6–32.1	
Lifetime IPV^a^	No	184 (32.5)	28.7–36.4	0.001	88 (15.6)	12.8–18.8	0.013
	Yes	220 (42.4)	38.2–46.7		111 (21.4)	18.1–25.2	
IPV during the pandemic^a^	No	303 (35.4)	32.3–38.7	0.021	150 (17.6)	15.2–20.3	0.199
	Yes	101 (43.7)	37.5–50.2		49 (21.3)	16.5–27.1	
Number of intimate partner violence during lifetime^a^	No violence	184 (32.5)	28.7–36.4	0.002	88 (15.6)	12.8–18.8	0.010
	One type of violence	91 (41.0)	34.7–47.6		46 (20.7)	15.9–26.6	
	Two types of violence	75 (47.8)	40.1–55.6		42 (26.8)	20.4–34.2	
	Three types of violence	54 (38.6)	30.9–46.9		23 (16.6)	11.2–23.7	
Number of intimate partner violence during the pandemic^a^	No violence	303 (35.4)	32.3–38.7	0.058	150 (17.6)	15.2–20.3	0.377
	One type of violence	57 (45.2)	36.8–54.0		28 (22.4)	15.9–30.5	
	Two types of violence	26 (48.2)	35.2–61.3		13 (24.1)	14.5–37.2	
	Three types of violence	18 (35.3)	23.5–49.2		08 (16.6)	8.0–28.4	

N, absolute frequency; %, relative frequency; 95% CI, 95% confidence interval. The bivariate analysis employed Pearson's chi-square test (*χ*^2^) or Fisher's exact test, as appropriate.

a Lifetime psychological, physical, and sexual violence.

Women's age range and the prevalence of lifetime use of sleep-inducing medicines increased after adjusting for confounders, 35% higher among those aged 40–59 years (95% CI: 1.12–1.64) and 53% higher among those aged 60 years and above (95% CI: 1.25–1.87) compared to younger women (18–39 years). Furthermore, having used some sleep-inducing medicine was more frequent among participants who are not in a marital relationship (PR: 1.22; 95% CI: 1.01–1.47).

Also, women who experienced childhood sexual violence were 1.51 times more likely to have used sleep-inducing medicine in their lifetime. Similarly, the group that reported having suffered IPV in their lifetime revealed a higher frequency of using this substance (PR: 1.29; 95% CI: 1.10–1.51). Finally, women with an IPV history during the pandemic were 22% (95% CI 1.03–1.45) more likely to have used sleep-inducing medicines, and experiencing one or two types of IPV during lifetime was associated with a higher use of sleep-inducing medicines (28% and 42%, respectively) (Table 3).

**Table 3:** Crude and adjusted analysis of the association between socioeconomic characteristics and experiences of violence with lifetime use of sleep-inducing medicines among women residing in Vitória, Espírito Santo, Brazil, 2022.

Variable	Category	Crude PR	95% confidence interval (95% CI)	*p-value*	Adjusted PR	(95% CI)	*p-value*
Age group (years)	18–39	*Ref.*		<0.001	Ref.		0.002
	40–59	1.33	1.10–1.61		1.35	1.12–1.64	
	60 and above	1.50	1.23–1.83		1.53	1.25–1.87	
Ethnicity/skin color	White people	*Ref.*		0.088	Ref.		0.130
	Black people	*1.00*	0.81–1.23		*1.05*	0.86–1.30	
	Brown people	*0.82*	0.69–0.98		*0.86*	0.71–1.01	
	Other	0.65	0.32–1.30		0.70	0.35–1.39	
In a relationship	Yes	*Ref.*		0.111	*Ref.*		0.037
	No	*1.17*	0.98–1.40		1.22	1.01–1.47	
Childhood sexual violence	No	*Ref.*		<0.001	*Ref.*		<0.001
	Yes	1.44	1.19–1.76		1.51	1.25–1.84	
Lifetime IPV^a^	No	*Ref.*		0.001	*Ref.*		0.002
	Yes	1.31	1.12–1.53		1.29	1.10–1.51	
IPV during the pandemic^a^	No	Ref.		0.017	*Ref.*		0.023
	Yes	1.23	1.04–1.47		1.22	1.03–1.45	
Number of intimate partner violence during lifetime^a^	No violence	*Ref.*		0.002	*Ref.*		0.006
	One type of violence	1.26	1.04–1.54		1.28	1.05–1.56	
	Two types of violence	1.47	1.20–1.80		1.42	1.15–1.78	
	Three types of violence	1.19	0.93–1.51		1.15	0.90–1.46	
Number of intimate partner violence during the pandemic^a^	No violence	*Ref.*		0.039	*Ref.*		0.052
	One type of violence	1.28	1.03–1.58		1.28	1.04–1.58	
	Two types of violence	1.36	1.02–1.82		1.31	0.98–1.75	
	Three types of violence	1.00	0.68–1.46		0.98	0.67–1.43	

Adjustment = Model 1: Crude (without adjustment); Model 2: Adjustment for sociodemographic factors; Model 3: Model 2 + adjustment for childhood sexual violence experience; Model 4: Model 3 + experiences of intimate partner violence (these experiences were adjusted separately from one another); IPV, intimate partner violence; PR, prevalence ratio; 95% CI, 95% confidence interval. The multivariate analysis employed Poisson regression with robust variance to estimate the prevalence ratio.

a Psychological, physical, and sexual violence perpetrated by an intimate partner during lifetime.

Regarding the current use of sleep-inducing medicines, we observed that women aged 40–59 years showed an 81% higher prevalence (95% CI 1.29–2.55) and those aged 60 and above showed a 2.53 times higher prevalence (1.80–3.57) than younger women. Regarding ethnicity/skin color, white women had a 58% higher prevalence (95% CI 1.18–2.11) of current medicine use than brown women, and those not in a marital relationship showed a 1.35 times higher prevalence (95% CI 1.01–1.82) of current use of sleep-inducing medicines. Regarding experiences of violence, we identified that exposure to childhood sexual violence was associated with the current use of sleep-inducing medicines (PR 1.55; 95% CI 1.11–2.17) and was also more prevalent among women who suffered one type (PR 1.41; 95% CI 1.94) or two types of IPV (PR 1.78; 95% CI 1.79) (Table 4).

**Table 4:** Crude and adjusted analysis of the association between socioeconomic characteristics and experiences of violence with the current use of sleep-inducing medicines among women residing in Vitória, Espírito Santo, Brazil, 2022.

Variable	Category	Crude PR	95% confidence interval (95% CI)	*p*-value	Adjusted PR	(95% CI)	*p*-value
Age group (years)	18–39	*Ref.*		<0.001	Ref.		<0.001
	40–59	1.79	1.27–2.52		1.81	1.29–2.55	
	60 and above	2.64	1.88–3.70		2.53	1.80–3.57	
Ethnicity/skin color	White people	*1.73*	1.30–2.31	0.002	1.58	1.18–2.11	0.021
	Black people	*1.27*	0.86–1.87		*1.32*	0.90–1.93	
	Other	0.95	0.32–2.80		*1.03*	0.37–2.87	
	Brown people	*Ref.*			Ref.		
In a relationship	Yes	*Ref.*		0.203	*Ref.*		0.046
	No	*1.22*	0.90–1.64		1.35	1.01–1.82	
Childhood sexual violence	No	*Ref.*		0.127	*Ref.*		0.011
	Yes	1.32	0.92–1.88		1.55	1.11–2.17	
Lifetime IPV^a^	No	*Ref.*		0.014	*Ref.*		0.005
	Yes	1.37	1.07–1.77		1.46	1.12–1.90	
IPV during the pandemic^a^	No	Ref.		0.194	*Ref.*		0.081
	Yes	1.21	0.91–1.61		1.29	0.97–1.73	
Number of intimate partner violence during lifetime	No violence	*Ref.*		0.008	*Ref.*		0.006
	One type of violence	1.32	0.96–1.83		1.41	1.02–1.94	
	Two types of violence	1.71	1.24–2.37		1.78	1.27–2.49	
	Three types of violence	1.06	0.70–1.61		1.17	0.76–1.79	
Number of intimate partner violence during the pandemic^a^	No violence	*Ref.*		0.361	*Ref.*		0.267
	One type of violence	1.27	0.89–1.82		1.31	0.93–1.86	
	Two types of violence	1.38	0.93–2.24		1.45	0.87–2.40	
	Three types of violence	0.89	0.46–1.71		1.05	0.54–2.02	

Adjustment = Model 1: Crude (without adjustment); Model 2: Adjustment for sociodemographic factors; Model 3: Model 2 + adjustment for childhood sexual violence experience; Model 4: Model 3 + experiences of intimate partner violence (these experiences were adjusted separately from one another); IPV, intimate partner violence; PR, prevalence ratio; 95% CI, 95% confidence interval. The multivariate analysis employed Poisson regression with robust variance to estimate the prevalence ratio.

a Psychological, physical, and sexual violence perpetrated by an intimate partner during lifetime.

## 4 Discussion

The prevalence of lifetime use of sleep-inducing medicines among women residing in Vitória was 37.2% (95% CI 34.4–40.1), and 18.4% (95% CI 16.2–20.8) for current use, indicating significant consumption of these medicines in the studied population. These findings show a pattern similar to that observed by Leite et al. (20), who identified prevalence levels of 45.9% for lifetime use and 18.5% for current use among women attended in Primary Care in the same municipality in 2014.

After adjusting for confounding variables, we observed that the prevalence of lifetime use of sleep-inducing medicines increased with women's age. It was 35% higher among those aged 40–59 years (95% CI 1.12–1.64) and 53% higher among those aged 60 years and above (95% CI 1.25–1.87) compared to younger women (18–39 years). These results are consistent with national findings. Leite et al. (20) also observed higher prevalence levels among older women and concluded that the use of sleep inducers is primarily concentrated in advanced age groups. Another population study with Brazilian older adults revealed that the use of benzodiazepines is significantly higher among seniors, reinforcing the increasing pattern with age (21).

Furthermore, when analyzing Brazilian adults, Araújo et al. (11) identified that poorer sleep quality, comorbidities, and psychosocial factors substantially increased the use of sleep-inducing medicines, elements that escalate with aging and justify the gradient found. Similar results were observed in another national study that showed that multimorbidity (presence of two or more chronic conditions in the same individual) patterns among women significantly increase the likelihood of using sleep-inducing medicines, especially among older women (22). Internationally, Montes-Castrejon et al. (23) found that benzodiazepine use is considerably more frequent in older adults, highlighting that prolonged use accumulates with age due to a greater burden of symptoms and continued prescriptions.

Another finding indicated that white women had a 58% higher prevalence of current use of sleep-inducing medicines than brown women (95% CI 1.18–2.11). This pattern suggests structural inequalities in access to and use of psychotropics according to ethnicity/skin color. In Brazil, population studies indicate that white persons tend to have greater access to health services, a higher probability of receiving prescriptions, and greater use of psychotropics, including benzodiazepines and hypnotics (11, 22). International evidence corroborates this finding: an analysis of health insurance data in the United States showed that white people are more likely to initiate and maintain the use of benzodiazepines compared to Black people and other racial groups (24).

These findings reinforce that racial differences in the use of sleep-inducing medicines reflect disparities in access to care, a greater recognition of symptoms, and a higher likelihood of prescription, which may explain the higher prevalence among white women in the present study. Furthermore, Leng et al. (25) showed that the use of sleep-inducing medicines is more prevalent among white people than among Black people in a cohort of older adults, and that frequent use of these medicines (five or more times per month) among white people is associated with additional risks, such as a higher risk (79%) of cognitive decline and dementia.

Regarding marital status and the use of sleep-inducing medicines, the highest prevalence was recorded among those who were not in any marital relationship, similar to other studies (11, 26) that indicate higher use among divorced people or those who do not live with a partner. Several factors may be related, such as that divorce is a stressful time in an adult's life, which can generate some adverse consequences (27), contributing to the higher use of sleep-inducing medicines.

The present study identified that women who experienced childhood sexual violence had a higher prevalence of current and lifetime sleep-inducing medicine use. In the same vein, Leite et al. (20) showed that the experience of childhood sexual violence was associated with lifetime use of sleep-inducing medicines, a PR of 1.33 (95% CI 1.13–1.56) among women in primary care in Vitória. According to Rai et al. (28), women exposed to sexual violence had a higher risk of developing anxiety, depression, insomnia, and post-traumatic stress disorder, conditions that frequently lead to the use of psychotropics and sleep inducers as a way to manage emotional distress. Thus, the association observed in the present study reflects the direct effect of sexual violence on mental illness and its repercussions on medicine use.

Women who reported IPV during the pandemic had a 22% higher incidence of lifetime use of sleep-inducing medicines (95% CI 1.03–1.45). This finding is consistent with Scoglio et al. (29), who show that the pandemic exacerbated IPV and its impacts on mental health; women exposed to IPV during the pandemic had poorer sleep quality, with a 21% increase in the likelihood of sleep disorders (OR = 1.21; 95% CI 1.16–1.26).

Furthermore, the findings of this research indicate that women who had already suffered IPV showed a higher prevalence of using sleep-inducing medicine during their lifetime (PR = 1.29; 95% CI 1.10–1.51). Matos et al. (30) identified that women exposed to intimate partner violence had higher odds of using sleep medicines (OR = 1.55; 95% CI 1.35–1.79), as in a study conducted by Leite et al. (20) (PR = 1.28; 95% CI 1.09–1.50).

Similarly, Stene et al. (31) observed OR = 2.28 (95% CI 1.73–3.00) for recent use of hypnotics among women who experienced intimate partner physical or sexual violence. Furthermore, the use of sleep-inducing medicines was associated with the number of lifetime IPV experiences. Similar findings were described by Leite et al. (20), who found an increase in the current use of sleep-inducing medicines among women who experienced three types of violence (PR = 2.39). According to Gallegos et al. (32), interpersonal violence, including IPV, causes significant changes in the physiological stress response, primarily through increased hypervigilance, fear, and autonomic activation. These mechanisms hinder sleep onset and preservation, favoring insomnia and fragmented sleep. Based on this dynamic, the hypothesis presented is that the use of sleep-inducing medicines among women who suffer IPV may occur as an attempt to attenuate this nocturnal physiological hyperactivation, seeking to induce relaxation that the body cannot achieve spontaneously while in a state of continuous alertness. Thus, the use of sleep inducers would be a strategy to mitigate the direct impacts of violence on the ability to sleep and recover energy (32).

From a clinical and public health perspective, the findings highlight the need for careful prescription and monitoring of sleep-inducing medicines among women, especially older individuals and those exposed to violence. The high prevalence suggests use beyond sleep disorders, reflecting underlying psychosocial and mental health conditions. Thus, comprehensive, multidisciplinary approaches and the integration of violence screening and mental health care into primary services are essential to promote safer and more equitable care (33).

A limitation of the present study is the data's cross-sectional nature, which hampers the establishment of causal relationships and relies on only one point in time for data collection. Furthermore, the use of a pre-existing database restricts the selection of variables available for analysis, as the researcher is limited to the information collected in the baseline study, which may result in the lack of factors that could expand and delve deeper into the study. In addition, the study may be subject to recall bias, particularly regarding the reporting of childhood sexual violence, as participants rely on memories of events that may have occurred many years earlier, especially among older adults. There is also the possibility of misclassification bias, since participants may not accurately recall or correctly identify the names or types of medications they have used. These factors should be considered when interpreting the findings.

## 5 Conclusion

We conclude that the lifetime use of sleep-inducing medicines was associated with socioeconomic characteristics and experiences of violence, namely, older age groups among women, participants who are not in a con-marital relationship, women who experienced childhood sexual violence, who have already suffered IPV during their lifetime, who have a history of IPV during the pandemic, and suffering lifetime IPV.

We also observed that current use of sleep-inducing medicines was associated with socioeconomic characteristics and experiences of violence, namely, being 40 or older, white people, not being in a marital relationship, having suffered childhood sexual violence, and being a woman who suffered IPV.

The findings reinforce that socioeconomic profile and exposure to violence are related to increased odds of using sleep-inducing medicines. Therefore, professionals can increase sensitivity in their assessments to evaluate the use of this type of medicine, which requires careful monitoring due to the potential for complications when misused. This context demands attention in public policies and health interventions.

## Data Availability

The raw data supporting the conclusions of this article will be made available by the authors, without undue reservation.

## References

[1] BRASIL. Lei Maria Da Penha, 2006. Brasília: Senado Federal, Subsecretaria de Edições Técnicas (2011). Available online at: https://www.planalto.gov.br/ccivil_03/_ato2004-2006/2006/lei/l11340.htm (Accessed December 20, 2025).

[2] Brasil. Relatório Anual Socioeconômico Da Mulher: RASEAM. Brasília: Observatório Brasil da Igualdade de Gênero/Mulheres (2025). Available online at: https://www.gov.br/mulheres/pt-br/central-de-conteudos/publicacoes/raseam-2025.pdf (Accessed December 10, 2025).

[3] Santos A Monteiro C Feitosa C Veloso C Nogueira L Andrade E. Types of non-psychotic mental disorders in adult women who suffered intimate partner violence. An integrative review. Rev Esc Enferm USP. (2018) 52:e03328. 10.1590/s1980-220x2017030203328 29846484

[4] Diniz NMF Gesteira SMA Lopes RLM Mota RS Pérez BAG Gomes NP. Voluntary abortion and domestic violence among women attended at a public maternity hospital of Salvador-BA. Rev Bras Enferm. (2011) 64(6):1010–5. 10.1590/S0034-71672011000600004 22664597

[5] Leite MG Rodrigues DP Sousa AAS Melo LPT Fialho AVM. Sentimentos advindos da maternidade: revelações de um grupo de gestantes. Psicol Em Estudo. (2014) 19:115–24. 10.1590/1413-7372189590011

[6] Silva S França M Marques L. Women and mental health: reflections from the bibliography and an experience report. Rev PsicoFAE. (2023) 12:2. 10.55388/psicofae.v12n2.432

[7] Pigeon WR He H Cerulli C Richards H Perlis M Caine E. Sleep disturbances and their association with mental health among women exposed to intimate partner violence. J Womens Health (Larchmt.). (2011) 20:12. 10.1089/jwh.2011.2781 PMC3236986 21988551

[8] Campbell JC Lewandowski LA. Mental and physical health effects of intimate partner violence on women and children. Psychiatr Clin North Am. (1997) 20:2. 10.1016/s0193-953x(05)70317-8 9196919

[9] Campbell JC. Health consequences of intimate partner violence. Lancet. (2002) 359:1331–6. 10.1016/s0140-6736(02)08336-8 11965295

[10] Pitanga I Neto J Cunha J Rodrigues C Moret M Lucas D Manejo do distúrbio do sono: uma revisão integrativa. Braz J Implantol Health Sci. (2024) 6(6):1135–44. 10.36557/2674-8169.2024v6n6p1135-1144

[11] Araújo MFS Souza TA Medeiros AA Souza JC Barbosa IR. Fatores associados aos problemas de sono e ao uso de medicação para dormir em brasileiros. Rev Saude Publica. (2022) 56:68. 10.11606/s1518-8787.2022056004088 35894405 PMC9337849

[12] World Health Organization (WHO). The Role of the Pharmacist in Self Care and Self-Medication. Dpt. of Essential Drugs and Other Medicines (1998). Available online at: https://iris.who.int/server/api/core/bitstreams/b5e735c5-7218-4230-bb04-aa37ab08c444/content (Accessed November 22, 2025).

[13] Leal RP Miranda LR Santos GB. Risks of self-medicine in young people with insomnia. Res Soc Dev. (2024) 13(9):e7313946721. 10.33448/rsd-v13i9.46721

[14] Wilson S Anderson K Baldwin D Dijk DJ Espie A Espie C British Association for Psychopharmacology consensus statement on evidence-based treatment of insomnia, parasomnias and circadian rhythm disorders: an update. J Psychopharmacol. (2019) 33:8. 10.1177/0269881119855343 31271339

[15] Pontes CAL Silveira LC. Benzodiazepine abuse among women: what does this phenomenon (un)veil? Sanare (Sobral, Online). (2017) 16:01. https://sanare.emnuvens.com.br/sanare/article/view/1089

[16] Instituto Brasileiro de Geografia e Estatística (IBGE). Estados: Espírito Santo: Censo 2010 (2022). Available online at: https://cidades.ibge.gov.br/brasil/es/panorama (Accessed July 20, 2025).

[17] Leite FMC Venturin B Ribeiro LEP Silva RDP Alves ML Wehrmeister FC Intimate partner violence against women during COVID-19: a population-based study in Vitória, State of Espírito Santo, Brazil. PLoS One. (2023) 18(12):e0295340. 10.1371/journal.pone.0295340 38117789 PMC10732366

[18] Schraiber LB Latorre MRDO França I Jr Segri NJ d'Oliveira AFPL. Validity of the WHO VAW study instrument for estimating gender-based violence against women. Rev Saude Publica. (2010) 44:658–66. 10.1590/s0034-89102010000400009 20676557

[19] García-Moreno C Jansen H Ellsberg M Heise L Watts C. WHO Multi-Country Study on Women's Health and Domestic Violence against Women: Initial Results on Prevalence, Health Outcomes and Women's Responses (2005). Available online at: https://onvg.fcsh.unl.pt/wp-content/uploads/sites/31/2019/11/924159358X_eng.pdf (Accessed October 20, 2025). 10.1126/science.1121400 16311321

[20] Leite FMC Xavier JCS Silva RP Wandekoken KD Tavares FL Amorim MHC. Prevalence and factors associated with the use of sleep-inducing medicine among women receiving primary health care: a cross-sectional study in Vitória, Espírito Santo, Brazil, 2014. Epidemiol Serv Saúde (Online). (2022) 31(1):e2021347. 10.1590/S1679-49742022000100016 35475997

[21] Freire M Silva B Bertoldi A Fontanella A Mengue S Ramos L Benzodiazepines utilization in Brazilian older adults: a population-based study. Rev Saude Publica. (2022) 56:10. 10.11606/s1518-8787.2022056003740 35319670 PMC8926397

[22] Oliveira M Schmidt M Sturmer J Franken D Costa J Olinto M Multimorbidity patterns as predictors of sleeping medicine use: a population-based study in women in southern Brazil. Rev Bras Epidemiol. (2024) 27:e240056. 10.1590/1980-549720240056 39699475 PMC11656498

[23] Montes-Castrejon A Moncayo-Samperio LG Flores-Ramos M. Benzodiazepine consumption, functionality, cognition, and somnolence in older adults at a tertiary care psychiatric hospital in Mexico City. Cureus. (2024) 16(1):e53252. 10.7759/cureus.53252 38298301 PMC10827568

[24] Chirica M Adams S Quinn P Meraz R Rickert M Sidorchuk A Psychiatric and racial/ethnic differences in incident and long-term benzodiazepine use: a commercial healthcare claims study. J Psychiatr Res. (2025) 184:155–62. 10.1016/j.jpsychires.2025.02.065 40049122 PMC11975480

[25] Leng Y Stone KL Yaffe K. Race differences in the association between sleep medicine use and risk of dementia. J Alzheimers Dis. (2023) 91(3):1133–9. 10.3233/JAD-221006 36565126 PMC10153591

[26] Seixas BV. Prevalence and factors associated with use of sleeping pills among older adults in Brazil. Int J Pharm Pract. (2021) 29:235–44. 10.1093/ijpp/riab003 33793814

[27] Lamela D. Development after divorce as a strategy of human growth. Rev Bras Crescimento Desenvolvimento Hum. (2009) 114–21. https://pepsic.bvsalud.org/scielo.php?script=sci_arttext&pid=S0104-12822009000100012

[28] Rai R. Sexual violence and poor mental health of women: an exploratory study of Uttar Pradesh, India. Clin Epidemiol Glob Health. (2020) 8(1):194–8. 10.1016/j.cegh.2019.06.013

[29] Scoglio A Zhu Y Lawn R Murchland A Sampson L Rich-Edwards J Intimate partner violence, mental health symptoms, and modifiable health factors in women during the COVID-19 pandemic in the US. JAMA Netw Open. (2023) 6:3. 10.1001/jamanetworkopen.2023.2977 PMC10015312 36917107

[30] Matos M Gonçalves M. Sleep and women intimate partner victimization: prevalence, effects and good practices in health care settings. Sleep Sci. (2019) 12(1):35–42. 10.5935/1984-0063.20190057 31105893 PMC6508940

[31] Stene LE Dyb G Jacobsen GW Schei B. Psychotropic drug use among women exposed to intimate partner violence: a population-based study. Scand J Public Health. (2010) 38(Suppl 5):88–95. 10.1177/1403494810382815 21062843

[32] Gallegos AM Trabold N Cerulli C Pigeon WR. Sleep and interpersonal violence: a systematic review. TVA. (2021) 22(2):359–69. 10.1177/1524838019852633 31131736

[33] Fu M Li C Zhou X Gong Z Zhu Y Ding Y Psychotropic medication prescribing for patients with insomnia comorbid with depressive or anxiety disorders in primary healthcare facilities in Beijing. BJPsych Open. (2026) 12(2):e55. 10.1192/bjo.2025.10967 41640068 PMC12926887

